# Plasma Concentration Rise after the Intramuscular Administration of High Dose Medetomidine (0.13 mg/kg) for Semen Collection in Cats

**DOI:** 10.3390/vetsci7010017

**Published:** 2020-02-03

**Authors:** Noemi Romagnoli, Carlotta Lambertini, Daniele Zambelli, Marco Cunto, Giulia Ballotta, Andrea Barbarossa

**Affiliations:** Department of Veterinary Medical Sciences, Alma Mater Studiorum-University of Bologna, Via Tolara di Sopra 50, Ozzano Emilia, 40064 Bologna, Italy; noemi.romagnoli@unibo.it (N.R.); daniele.zambelli@unibo.it (D.Z.); marco.cunto@unibo.it (M.C.); giulia.ballotta2@unibo.it (G.B.); andrea.barbarossa@unibo.it (A.B.)

**Keywords:** anaesthesia, feline, medetomidine, plasma concentration, semen collection

## Abstract

High dose medetomidine 0.13 mg/kg can be used for semen collection in cats with variable results in terms of quantity and quality. Therefore, a variation in terms of distribution and elimination among patients has been hypothesised. The aim of the study was to characterise the pharmacokinetics of medetomidine (0.13 mg/kg) administered intramuscularly (IM) in healthy male cats. Eighteen male cats undergoing castration were included, and medetomidine (0.13 mg/kg) was administered IM. Venous blood samples were collected at 20, 30, 40, 50, 60, 75 and 90 minutes after medetomidine administration. Before orchiectomy, at T20, sperm collection was attempted. Plasma medetomidine concentrations were determined by liquid chromatography/mass spectrometry analysis. Semen collection was successful in 15/18 cats. The medetomidine plasma concentration following the IM administration of a bolus was best described using a non-compartment model. Time of maximum concentration was observed at 40 minutes (range 20–90); maximum concentration was 32.8 ng/mL (range 26.8–51.2). The median apparent clearance was 11.9 mL/kg/minute (range 0.7–43.8). In conclusion, medetomidine administered IM at 0.13 mg/kg reached its peak plasma concentration slowly and with variability among patients. In addition, it was characterised by low total body clearance probably due to the cardiovascular alterations associated with medetomidine administration.

## 1. Introduction

Medetomidine is an alpha_2_-adrenoceptor agonist commonly used in veterinary anaesthesia to obtain sedation, analgesia and muscle relaxation. In addition, in male cats and in wild felids, the administration of medetomidine has also been included in the protocol for semen collection after pharmacological induction (Ur.Ca.P.I.) [1,2,3]. This technique was first described in tomcats to allow semen collection by means of urethral catheterisation after the administration of high dose medetomidine (0.13–0.14 mg/kg) [4]. In another study, the authors demonstrated that the semen collected using the Ur.Ca.P.I. technique was characterised by a lower volume but a higher sperm concentration when compared with that collected by electroejaculation [4]. The mechanisms of action by which medetomidine determines the sperm release into the urethra is still to be determined. Medetomidine is characterized by a high affinity for alpha_2_-adrenoceptors but it maintains affinity also for alpha_1_-adrenoceptors. Studies in humans and in animal models have described that alpha_1_ and alpa_2_ adrenoceptors are expressed in the urethra where they regulate the urethral contraction [5,6]. In addition, in rats, the alpha_1_-adrenoceptor agonists induce the contraction of the vas deferens, and the alpha_1A_-adrenoceptors are the adrenoceptor subtype involved in this mechanism [7]. 

In clinical practice, the doses of medetomidine used for obtaining sedation in cats range from 0.005 to 0.08 mg/kg [8]. However, when the Ur.Ca.P.I. technique was attempted in cats using a lower dose of medetomidine (0.05 mg/kg), urethral catheterisation allowed the collection of semen characterised by a lower volume, a lower sperm concentration, and a lower percentage of motility and progressive motility as compared with that obtained after the administration of the full dose [9]. 

Dose-dependent cardiovascular side effects have commonly been reported after the administration of alpha_2_-agonists [10,11,12]. However, in healthy cats, the administration of high dose medetomidine resulted in echocardiographic alterations similar to those previously reported after the administration of lower dosages (0.02 mg/kg) [13,14].

When the Ur.Ca.P.I. technique is applied in cats, even if the same dose of medetomidine is administered (0.13 mg/kg IM), the effect on semen collection, in terms of quantity and quality, is variable among cats. The authors hypothesised a variation of the pharmacokinetic parameters of intramuscular (IM)-administered medetomidine in cats in terms of absorption, distribution and elimination. Therefore, the aim of this study was to investigate the plasmatic concentration and the pharmacokinetic parameters of medetomidine after IM administration in male cats.

## 2. Materials and Methods

### 2.1. Animals

Eighteen privately owned adult male cats admitted to the Veterinary Teaching Hospital of the University of Bologna and undergoing orchiectomy were included in the study. Their mean weight was 3.9 ± 0.8 kg and their mean age was 24.2 ± 14.4 months. Before the procedure, the cats underwent a physical examination and they were graded by the anaesthetist according to the American Society of Anaesthesiologist (ASA) Classification. Only those cats considered healthy with no detectable disease and therefore classified in class ASA I, were included in the study. Before the beginning of the study, informed consent was obtained from the owners.

The study was conducted with the approval of the Ethics Committee of the University of Bologna in accordance with the Directive 2010/63/UE of the European Parliament and Council, adopted by the Italian Government (D.L. 4/3/2014 n.26) Prot. n. 211643.

### 2.2. Anaesthesia and Semen Collection

Food was withheld overnight; access to water was provided until two hours before the procedure. On the day of the surgery, the cats were premedicated with medetomidine 0.13 mg/kg (Sedastart, ESTEVE S.p.A) administered IM in the gluteal muscle. As soon as the cats achieved lateral recumbency a 20G intravenous (IV) catheter was placed in the jugular vein after aseptic preparation of the area. This catheter was used for blood collection for the pharmacokinetic study. An adjunctive 22G IV catheter was placed into the cephalic vein for fluid and drug administration. Lactated Ringer’s solution (Ringer lattato, ACME; Reggio Emilia, Italy) was administered IV throughout anaesthesia at the rate of 5 mL/kg/hour and oxygen was provided throughout the procedure using a face mask. Intraoperative monitoring consisted of evaluating heart rate (HR) and haemoglobin oxygen saturation (SpO2) using a pulse-oximeter, respiratory rate (*f*_R_) by visual inspection of the thorax, pulse character by digital palpation of the femoral pulse, mucous membrane appearance by visual inspection and body temperature using a thermometer. Body temperature was maintained approximately constant using a forced air warming blanket when necessary (Bair Hugger, 3M, Berkshire, UK).

Venous blood samples for the pharmacokinetic evaluation were collected 20 (T20), 30 (T30), 40 (T40), 50 (T50), 60 (T60), 75 (T75) and 90 (T90) minutes after medetomidine administration in k3-EDTA vials from the jugular vein catheter. Twenty minutes after medetomidine administration (T20), semen collection was attempted as previously described [4]. In brief, a 3F 11 cm long urinary catheter ((Portex® Jackson Cat Catheter, St Paul, MN, USA) was inserted into the urethra in order to collect the semen released into the urethra itself. 

After semen collection, an experienced surgeon performed the orchiectomy. 

All the cats were monitored during the surgical procedures for signs of insufficient anaesthetic depth. Purposeful movements were considered indicative of inadequate anaesthetic depth and, in this case, isoflurane in oxygen was provided by mask.

An increase in HR values greater than 20% as compared with the values recorded before the application of the stimulus were considered indicative of inadequate analgesia. Cats requiring additional analgesia were excluded from the study.

After the collection of the last blood sample and 90 minutes after medetomidine administration, atipamezole (Sedastop, ESTEVE S.p.A) 0.3 mg/kg was administered IM to reverse sedation. In addition, all the cats received meloxicam (Metacam, Boehringer Ingelheim, Germany) 0.1 mg/kg subcutaneously.

All the cats were monitored throughout the recovery period and were discharged from the hospital when completely awake.

### 2.3. Semen Collection and Evaluation

The semen evaluation was performed as previously described [9]. In details, after collection, the semen from each cat was collected in 0.5-mL Eppendorf tubes, and macroscopic and microscopic evaluations were performed. The sperm volume was determined using a variable volume pipette. The remaining evaluations were performed on diluted samples, obtained by adding 18 μL of Tris–glucose–citrate to 2 μL of sperm. The Motility (0%–100%) and the movement score (scale 0–5) were assessed subjectively using a Phase contrast microscope (Axiolab, Zeiss, Oberkochen, Germany). The sperm concentration was assessed using a Bürker chamber.

### 2.4. Plasma Sample Analysis

The plasma samples were analysed using a previously published procedure [15], with slight modifications. After being thawed at room temperature, 250 µL of plasma were transferred to a microtube, followed by 500 µL of a 9:1 acetonitrile:1 M acetic acid aqueous solution containing 25 ng/mL of deuterated internal standard (medetomidine-d3). The sample was then vortex mixed for 2.0 min, refrigerated for 30.0 min and centrifuged for 10.0 min at 1500 ×*g* at 5°C. Finally, 150 µL of supernatant were transferred into an LC vial containing 150 µL of 0.2% formic acid in water.

A 10.0 µL aliquot of the sample was then injected into the UPLC-MS/MS system, consisting of a Waters Acquity ultra performance liquid chromatography (UPLC) binary pump, equipped with an Acquity BEH C18 (50 × 2.1 mm, 1.7 μm) column and coupled to a Quattro Premier XE triple quadrupole mass spectrometer (Waters, Milford, MA, USA). The column temperature was set at 35 °C, and the mobile phase consisted of a gradient mixture of 0.1% formic acid aqueous solution and acetonitrile flowing at 0.4 mL/minute. The mass spectrometer operated in positive electrospray ionisation mode (ESI+), with voltage capillary set at 3.0 kV and source temperature at 120 °C. The specific transitions monitored for medetomidine and the internal standard were 201.0 > 95.1 *m/z* and 204.1 > 98.3 *m/z*, respectively.

The analytical method was validated in accordance with the EMEA/CHMP/EWP/192217/2009 guidelines before being applied to the samples collected, showing good linearity (R^2^ > 0.99) in the 1–1000 ng/mL range as well as satisfying interday and intraday accuracy and precision (< 15%, at three different concentrations).

### 2.5. Statistical Analysis

The statistical analysis was carried out using MedCalc computer software (MedCalc Statistical Software version 14.8.1). Data were evaluated for normality using a Shapiro–Wilk test. The heart rates and *f*Rs collected at each time point were compared with the baseline values using a Student’s t-test. A *p* < 0.05 was considered statistically significant.

Demographic data, the results of sperm evaluation, HR, *f*R and SpO2 are reported as mean ± standard deviation (SD); pharmacokinetic data are reported as median and range and interquartile range (IQR).

All pharmacokinetic parameters were determined using WinNonlin 6.4 (Pharsight Corporation, Mountain View, CA, USA). The individual plasma concentration-versus-time curves were fitted, and the best non-compartment model was determined by application of the Akaike information criterion [16]. The following pharmacokinetic parameters were evaluated for medetomidine for each cat: the time of maximum concentration (Tmax), the maximum concentration (Cmax), the area under the time-concentration curve from T0 to the last sample (AUC), the apparent clearance (Cl), the mean residence time (MRT), the volume of distribution (Vd).

## 3. Results

Eighteen male cats were included in the study. The protocol used for sedation allowed obtaining the lateral recumbency in all cats even if they were still responsive to minor tactile stimuli. Vomiting was observed in four cats (22%). The Ur.Ca.P.I. technique produced successful semen collection in 15/18 cats at T20. In three cats the sample collected did not contain spermatozoa. The mean volume of semen collected was 12.1 ± 4.4 µl with a mean concentration of 1071.4 ± 945.3 × 10^6^ spermatozoa/mL and a mean total number of spermatozoa of 11.7 ± 10.7 × 10^6^. The spermatozoa of the samples collected had a mean motility of 52.5 ± 22.2% and a mean movement score of 3.9 ± 0.9.

Isoflurane was administered to all the cats in order to perform the orchiectomy; the mean isoflurane dose was 1.5 ± 0.6%. Isoflurane was administered for a maximum of fifteen minutes from the beginning of the surgery within T30 and T50. The heart rate, *f*R and SpO_2_ recorded at each time point are summarised in Table 1. Medetomidine administration resulted in a statistically significant decrease in HR at all time points when compared with baseline. The *f*R did not change significantly when compared with the baseline values. All the cats maintained a normal or slightly pale mucous membrane throughout the study period, and the SpO_2_ remained within the normal limits.

The medetomidine plasma concentration following IM administration of a bolus was best described using a non-compartment model. The pharmacokinetic results are summarised in Table 2 and the median plasmatic concentrations at each time point are reported in Table 3. The median tmax was observed at 40 minutes (range 20–90) and the median Cmax was 32.8 ng/mL (range 26.8–51.2). The median AUC was 2240 (1759–3340) ng minute/mL and the median Vd was 3724.2 (2431.5–4806.4) mL/kg. The median Cl was 11.9 (0.7–43.8) mL/kg/minute. The mean residence time was 50.8 minutes (46.1–55.8). Recovery was uneventful for all cats and the owners did not report any abnormalities at home.

## 4. Discussion

In this study, the pharmacokinetics of high dose medetomidine (0.13 mg/kg) in isoflurane-anaesthetised cats was determined for the first time.

Medetomidine administered at 0.13 mg/kg in healthy male cats produced good sedation which was sufficient for obtaining immobilisation for catheter placement and for semen collection; however, castration could not be performed without the administration of isoflurane. Granholm and colleagues had previously reported that castration could be successfully performed only in one out of four cats sedated with medetomidine 0.08 mg/kg or an equipotent dose of dexmedetomidine [12]. In halothane anesthetized dogs undergoing laparoscopy, medetomidine premedication provided equipotent anaesthetic depth and analgesia compared with higher concentration of the inhalant anaesthetic drug [17]. However, when alpha_2_ agonists are administered in combination with opioids or dissociative anaesthetic drugs, their sedative effect is enhanced without clinically significant cardiovascular depression [18]. Dexmedetomidine is the active enantiomer of the racemic medetomidine and when administered at 0.04 mg/kg it induces analgesia in cats [19]. Nevertheless, in male cats undergoing castration, a lower dose of dexmedetomidine (0.025 mg/kg), if combined with ketamine and butorphanol or hydromorphone, provides sufficient anaesthesia to complete the surgical procedure [20]. However, when a different anaesthetic protocol is administered in cats for the UrCa.P.I. technique, worst results have already been reported in terms of quality of semen collected compared with those obtained after the administration of the full dose of medetomidine [9,21].

Vomiting was the most significant adverse effect related to the medetomidine administration observed in this study. For all cats, 12-hour fasting was recommended; however, vomiting had previously been reported in fasting cats sedated with an alpha_2_-agonist [12,22] due to its stimulation of the chemoreceptor trigger zone.

Sedation with medetomidine determined a significant decrease in HR when compared with baseline. The results in the present study are in accordance with previous studies in cats [10,11,12]. The cardiovascular effects of alpha_2_-agonists consist in bradycardia or brady-arrhythmias associated with a biphasic pressure response, and a significant reduction in the cardiac output and cardiac index [13]. The evaluation of the cardiovascular effects of 0.13 mg/kg of medetomidine was beyond the aim of this study; however, the hemodynamic effects of the same dose of medetomidine (0.13 mg/kg) in healthy cats have already been described in a previous study [14]. Based on echocardiographic evaluation, high dose medetomidine induced significant haemodynamic alterations in cats with an impairment in the systolic function [14] but the alterations were similar to those previously observed in cats receiving lower doses of medetomidine (0.02 mg/kg) and they were not clinically significant [13]. The rate of respiration was not significantly affected by the administration of medetomidine as previously reported by other authors [10].

Medetomidine is a lipophilic compound and, therefore, rapid absorption was expected after intramuscular administration. In non-compartment pharmacokinetic analysis, the absorption rate is described by the Cmax and tmax [23]. In the present study, medetomidine administration resulted in slow absorption after IM administration. In addition, large intersubjective variability in the medetomidine tmax was observed (range 20–90 minutes). Many factors influence drug absorption after IM administration: the site of injection, the depth of the injection but especially regional perfusion [24]. The IM injection was performed in the gluteal muscle in all the cats but differences in the regional perfusion cannot be excluded. Similar results were previously described by Salonen [25] after the IM administration of medetomidine in cats. In that study, the author observed the tmax within 15 and 30 minutes after the administration of medetomidine 80 mcg/kg in cats and dogs respectively [25]. A large tmax has been previously observed also after the IM administration of dexmedetomidine in cats. The administration of dexmedetomidine 25 mcg/kg IM resulted in a median tmax of 17.8 minutes (range: 2.6–44.9 minutes) [26] and the administration of a higher dose (40 mcg/kg) IM resulted in a tmax of 0.3 ± 0.25 hours (mean ± SD) [27]. Conversely, a higher dose of IM medetomidine (200 mcg/kg) in cats resulted in a shorter tmax (21.57 ± 10.05 minutes) [28].

Despite the slow absorption rate and the variability in the tmax, sedation was obtained within 5 minutes in all cats, and this result was in accordance with previous studies [11,12,28] even if the peak sedative effect could be observed even within 30 minutes after the IM administration [12]. Medetomidine rapidly diffuses into the brain; in rats, medetomidine rapidly reaches the central nervous system, and high levels of this drug can be found in the brain already at 5 minutes after the subcutaneous administration of a dose of 0.08 mg/kg [25]. The lack of difference in the sedation time which was observed in the present study despite the variability in tmax could be explained by hypothesising a ceiling effect in the sedative effect of medetomidine [28]. The correlation of the degree of sedation and the plasmatic concentration was not performed and was beyond the aim of the study. However, in the cats, the peak sedation was observed at a serum concentration of 77.7 ng/mL but, beyond a certain level, the sedative effect of medetomidine may be reduced or even reversed by means of the activation of alpha_1_-adrenoceptors [12].

Medetomidine is metabolised by the liver, and only a limited fraction of unchanged drug is excreted with faeces or urine [29]. In the present study, slow Cl of medetomidine was obtained, and it was higher when compared with that previously reported in cats (29.5 mL/kg/min) sedated with medetomidine 0.08 mg/kg [25]. The hepatic metabolism of drugs is dependent on hepatic blood flow [24,25]. Despite their significant reduction in CO, alpha_2_-agonists, when administered at doses commonly used in the clinical practice, do not provide a significant alteration in the hepatic blood flow [30]. However, in the present study a high dose of medetomidine was administered and the effects on the hepatic blood flow were not determined. In addition, isoflurane was administered to all the cats in order to perform the orchiectomy. Inhalant anaesthetic drugs alter organ perfusion, and the hepatic blood flow thus affecting the metabolism of drugs, clearance and volume of distribution [31,32]. Therefore, the pharmacokinetic results of the present study can be considered applicable only in isoflurane-anesthetised cats.

The quality of semen collected in the present study was similar, lower or higher compared with results previously reported [1,9,33,34]. Anyway, it can be considered in line with literature and differences observed can be due to variability of cats’ population between studies.

## 5. Conclusions

In conclusion, the results of the present study suggested that, after IM administration, medetomidine reaches peak plasmatic concentration slowly; in addition, inter-individual variability among cats may be expected. This might explain the differences observed in the results of the semen collection. Therefore, based on the range of the plasmatic concentration that was obtained in this study, when sperm collection was not feasible, additional attempts could be carried out up to 90 minutes after medetomidine administration. Additional studies are necessary to evaluate the alpha adrenergic receptor expression and distribution in the cat urethra, thereby clarifying the mechanism of action of medetomidine in determining semen collection.

Finally, although medetomidine administered alone at 0.13 mg/kg in cats provides sufficient sedation to collect the semen through urethral catheterization, it does not provide an adequate depth of anaesthesia to perform the orchiectomy and therefore the supplementation with inhalant anaesthetic agent maybe necessary if the surgery is to be performed. 

## Figures and Tables

**Table 1:** Monitoring parameters.

Parameters	T0	T20	T30	T40	T50	T60	T75	T90
HR (beats/min)	191.2 ± 16.8	101.1 ± 15.1 *	105.7 ± 15.4 *	109.6 ± 16.4 *	110.6 ± 11.1 *	105.3 ± 13.8 *	105.4 ± 9.5 *	115.7 ± 12.7 *
fR (breaths/min)	25.1 ± 12.6	44.2 ± 19.9	36.3 ± 10.5	40 ± 12.8	44.1 ± 19.7	35.1 ± 8.6	33.6 ± 10.5	26.1 ± 4.9
SpO_2_		98.3 ± 2.1	97.9 ± 1.6	99.3 ± 0.5	99.5 ± 0.7	97.25 ± 2.9	97.7 ± 0.6	98.2 ± 0.7

Heart rate (HR), respiratory rate (*f*_R_) and haemoglobin oxygen saturation (SpO_2_) after medetomidine intramuscular administration (0.13 mg/kg) in eighteen male cats. Data are reported as mean and standard deviation (SD). Data were collected at baseline (T0) and 20 (T20), 30 (T30), 40 (T40), 50 (T50), 60 (T60), 75 (T75) and 90 (T90) minutes after medetomidine administration. (*) statistically significant different from the baseline value (*p* < 0.05).

**Table 2:** Pharmacokinetic parameters.

Parameters	Values (median and range)	IQR
Time to reach peak concentration (minutes)	40 (20–90)	20–75
Peak concentration (ng/mL)	32.8 (26.8–51.2)	28.5–37.5
Area under the time-concentration curve (ng minute/mL)	2240 (1759–3340)	1967.6–2646.3
Volume of distribution (mL/kg)	3724.2 (2431.5–4806.4)	2584.6–4712.7
Apparent clearance (mL/kg/minute)	11.9 (0.7–43.8)	6.9–25.9
Mean residence time (minutes)	50.8 (46.1–55.8)	48–52.8

Pharmacokinetic parameters of medetomidine 0.13 mg/kg in eighteen male cats following intramuscular administration. Data are reported as median and range and interquartile range (IQR).

**Table 3:** Plasmatic concentration.

Time Points	Plasmatic Concentration (ng/mL)
0	0,0 (0–0)
20	27.9 (17–50.3)
30	28.2 (22–41.6)
40	28.7 (19.6–39.9)
50	28.8 (19.4–51.2)
60	28.5 (18.6–47.4)
75	28.6 (20.1–43.4)
90	27.7 (16.5–42.9)

Medetomidine plasmatic concentration at each time of sampling following the intramuscular administration of 0.13 mg/kg medetomidine in eighteen male cats. Data are reported as median and range.
